# Special Issue “Novel Chemical Tools for Targeted Cancer Therapy”

**DOI:** 10.3390/ijms25116044

**Published:** 2024-05-30

**Authors:** Alexander A. Shtil

**Affiliations:** Blokhin National Medical Research Center of Oncology, 24 Kashirskoye Shosse, 115522 Moscow, Russia; shtilaa@yahoo.com

When, in 2022, the *International Journal of Molecular Sciences* asked me to edit the Special Issue, I was quick to propose the title ‘Novel Chemical Tools for Targeted Cancer Therapy’. My idea was to invite a broad community of medicinal chemists, cancer biologists and perhaps clinicians to present their recent findings on the most perspective direction, namely, the concept of target, as a necessary prerequisite of today’s anticancer drug design. As a result of joint efforts of the researchers and the editorial team, the Special Issue was published (List of Contributions) with individual articles already acquiring visibility. I am sincerely grateful to the authors and the journal for this wonderful opportunity, valuable experience and new professional contacts worldwide.

Now, when it is time for the closing remarks, I share my considerations in repercussion of the Special Issue and my daily work. Indeed, the era of targeted anticancer treatment launched by the pioneering discovery of Imatinib (Gleevec^TM^) as an Abl tyrosine kinase inhibitor revolutionized the situation with chronic myelogenous leukemia (CML), a malignancy pathogenetically linked to the *BCR-ABL* gene rearrangement. Beyond its undoubtful significance for thousands of patients, this pivotal achievement proved a fundamental principle, that is, validation of the concept of drug targets, as well as the opportunity to rationally design the efficient drug based on knowledge about the target’s structure together with the skills in chemical engineering and conclusive testing of biological mechanisms. Over the subsequent three decades, dozens of targeted drugs have entered the practice or underwent clinical and/or preclinical trials.

However, this concept is a subject for critical reassessment. A comprehensive analysis has recently been presented in a pivotal article by A. Sadri [1]. The author performed a detailed and thoughtful study of the pros and cons of targeted drug design and demonstrated implicit limitations of the concept. To me, among the most crucial difficulties is an intrinsic redundancy of mechanisms of tumor initiation and progression, each unveiling in its due course. Therefore, targeting one particular mechanism may not be sufficient even if this mechanism is generally critical for tumor survival. Pursuing the example of CML, the initially successful targeting of BCR-ABL, rather selective as it used to be, becomes inefficient once the bypass pathways are developed or the target is mutated. Should we nevertheless focus on another selective agent when the primarily targeted drug failed? Are we aware why new generations of BCR-ABL inhibitors may not be efficacious given that the selectivity of kinase inhibitors, be they catalytic (nilotinib, ponatinib, dasatinib) or allosteric (asciminib), is arguable?

Thus, we must identify the target critical for the disease relapse. Most likely, such a target would differ across the patient cohort, making personalized therapy problematic. A plausible strategy is to broaden the principle, i.e., to inactivate more than one target and activate the initially silent (or less significant) death mechanism(s). Apparently, the newly identified target(s) should be druggable, a prerequisite for an additional level of complexity.

This approach does not contradict or downplay the idea of targeting. However, during disease progression, the ‘precision’ of this strategy weakens since too many factors become involved in the maintenance of uncontrolled tumor cell growth and pleiotropic resistance to therapeutic interventions. Perhaps CML is an infrequent example of the malignancy in which one mechanism remains predominant at disease onset as well as onwards; this mechanism is remarkably druggable. Therefore, it is not surprising that the principle of targeted drug design produced only ~one-tenth of clinical drugs [1]. The reason for this fact is not as much an insufficient knowledge in structural biology or synthetic obstacles, but the multiplicity and redundancy of biological regulation, leaving alone not less important issues of the shared role of a specific target in normal and tumor cells, the questionable selectivity, off-targeting, etc.

Most importantly, the use of the target-specific drug in the first line of therapy would select the cells for survival under this treatment. The secondary tumor would require an alternative treatment; the selectivity of further targeting becomes vague. How do we improve the efficacy of individual targeting and delay, if not prevent, the intractable relapse? Growing evidence of the epigenetic emergence of drug resistance has established a role of fine tuning the transcriptional machinery for rapid cell adaptation to cytotoxic stress [2]. At least two key circumstances favor the benefits of transcriptional reprogramming in anticancer therapy. First, some mechanisms of transcriptional reprogramming appeared to be successfully druggable with highly selective small molecular weight compounds, in particular, the cyclin-dependent kinases 8/19 that, together with their protein partners, comprise the module of the transcriptional Mediator complex [2]. Second, pharmacological inactivation of these enzymes is non-toxic in cell culture and tolerable by the adult organism. Taking advantage of these fortunate properties of transcriptional reprogramming, one may justify an approach to combinations of target-specific drugs. Indeed, selective inhibitors of the above-mentioned kinases prevented the survival of breast carcinoma cells treated with the estrogen signaling blocker fulvestrant [3]. Furthermore, the synergy with epidermal growth factor receptor antagonists, as well as circumvention of castration resistance in prostate cancer, demonstrated the efficacy of dual selective targeting of the tumor-specific mechanism and the transcription-regulating module which is generally tumor-independent but acts in concert with the antitumor drug [4,5]. In this scenario, the antitumor effect was achieved not via eschewing the principle of specific targeting but by ‘extending’ it to dual targeting, thereby keeping the validity of the concept. Still, transcriptional reprogramming is context-dependent; therefore, the applicability of its inhibition for tumor sensitization to the targeted anticancer drug should be tested in advance to avoid false negative interpretation.

Thus, identification of the proper situations is a key requirement for the therapeutic efficacy of targeted drugs. Obviously, this does not belittle this concept in general. The multiplicity of mechanisms in cancer dictates the inevitability of their identification for therapeutic intervention; in this sense, the mechanism-based, rationally designed chemical tools remain valuable. Finding the right applications for these instruments, as well as establishing the balance with the phenotype-based search [1], are the subject of future effort.
